# Synergy of the Inhibitory Action of Polyphenols Plus Vitamin C on Amyloid Fibril Formation: Case Study of Human Stefin B

**DOI:** 10.3390/antiox10091471

**Published:** 2021-09-15

**Authors:** Alma Jahić Mujkić, Magda Tušek Žnidarič, Selma Berbić, Eva Žerovnik

**Affiliations:** 1 Department of Biochemistry and Molecular and Structural Biology, Jožef Stefan Institute, Jamova 39, 1000 Ljubljana, Slovenia; 2 Department of Biochemistry, Faculty of Pharmacy, University of Tuzla, Univerzitetska 1, 75000 Tuzla, Bosnia and Herzegovina; alma.jahic@untz.ba (A.J.M.); selma.berbic@untz.ba (S.B.); 3 Department of Biotechnology and Systems Biology, National Institute of Biology, Večna pot 111, 1000 Ljubljana, Slovenia; magda.tusek.znidaric@nib.si; 4 Jožef Stefan International Postgraduate School, Jamova 39, 1000 Ljubljana, Slovenia

**Keywords:** protein aggregation, inhibition of amyloid fibril formation, polyphenols, curcumin, resveratrol, quercetin, ascorbic acid, vitamin C, synergistic effect of antioxidants, human stefin B, progressive myoclonus epilepsy of type 1—EPM1

## Abstract

In order to study how polyphenols and vitamin C (vitC) together affect protein aggregation to amyloid fibrils, we performed similar in vitro studies as before using stefin B as a model and a potentially amyloid-forming protein (it aggregates upon overexpression, under stressful conditions and some progressive myoclonus epilepsy of tape 1—EPM1-missense mutations). In addition to the chosen polyphenol, this time, we added a proven antioxidant concentration of 0.5 mM vitC into the fibrillation mixture and varied concentrations of resveratrol, quercetin, and curcumin. Synergy with vitC was observed with curcumin and quercetin.

## 1. Introduction

Understanding protein misfolding and amyloid fibril formation is essential to better understand and target protein misfolding diseases (PMDs), including neurodegenerative diseases, such as Alzheimer’s, Parkinson’s, and amyotrophic lateral sclerosis. In addition to stabilizing the native state or stable oligomer of a protein to undergo aggregation, the very process of amyloid formation can be modulated or inhibited at a certain step by small molecules.

As practically all proteins can be transformed into amyloid fibrils [1], one can use appropriate model proteins to study amyloidogenesis. When structural data on the process of oligomerization and amyloid formation of the chosen protein are available, even greater insight may be gained. Our case study protein, human stefin B, is a member of the superfamily of cystatins, cysteine protease inhibitors, and known amyloidogenic proteins. It has been shown to possess stable oligomers and prefibrillar aggregates [2,3], while avoiding the infectivity of prions or amyloid-beta.

Human stefin B has been used as an easy model protein to undergo amyloid fibril formation in slightly acidic conditions [2,4], even though it forms oligomers even at neutral pH [5]. The tetramer’s structure, containing two domain-swapped dimers, was solved [6]. Of interest, one Pro in loop No. 2, Pro74, was found in *cis*, and the *cis/trans* isomerization might play a role as a switch in amyloid-fibril formation, as shown by mutational analysis [7].

The hypothesis that some antioxidant substances may act as inhibitors of amyloid fibril formation is not new. This idea was already pointed out by Ono et al. [8] and Porat et al. [9], who studied the effects of curcumin and some other polyphenols on amyloid formation by amyloid-beta (Aβ) peptide and islet amyloid polypeptide (amylin). Numerous other reports on curcumin’s effects on protein aggregation can be found in the literature. From computational predictions [10] to experimental data [11], it has been proven that curcumin has a strong anti-amyloid effect. It prevents aggregation of Aβ peptide [11]; furthermore, because it crosses the blood-brain barrier (BBB), it also indirectly preserves/improves cognition in Alzheimer’s disease (AD) [12]. Curcumin’s effects on α-synuclein [13] and prion [14] fibril formation have also been observed. Derivatives [15] and nanoparticles [16] have been developped to improve its import into the brain. Interestingly, toxic Tau oligomers could be modulated to become less toxic by novel curcumin derivatives [17]. Some other polyphenols seem to prevent membrane damage and pore formation by the α-synuclein oligomers [18].

A number of epidemiological studies have shown that older people who are following a specific diet regime rich in natural antioxidants show lower incidence of AD and Parkinson’s disease (PD) [19,20]. In particular, polyphenols were found beneficial. Of note, natural polyphenolic compounds protect plants against UV radiation [21]. More and more in vitro and in vivo studies are being conducted showing that natural polyphenols inhibit amyloid fibril formation and/or destabilize already formed fibrils [22,23]. The mechanism underlying how these kinds of compounds work against fibril formation is not clear. There are indications that both anti-amyloidogenic and antioxidative effects work together, thereby giving even higher protection. A recent example is OleA, a polyphenol from olive oil, where the authors (from the Morozova-Roche and Bucciatini groups) studied S100A9 aggregation in the presence and absence of this compound and were able to explain its anti-amyloid and anti-oligomer effects. It even lowered membrane binding of S100A9 oligomers and elevated cytotoxicity [24]. Similarly, although likely via yet another mechanism, ascorbic acid—vitamin C (vitC) also has both anti-amyloid and anti-oxidant properties [25]. Such an action of vitC is limited to a range of lower concentrations and also depends on the protein. We showed before that 0.5 mM vitC is low enough concentration in the case of stefin B to inhibit amyloid fibril formation and not act in a pro-oxidant manner [26,27].

It is worth mentioning that our model protein stefin B was shown to bind Cu^2+^ [28]. The binding of the metal influenced protein aggregation and inhibited amyloid fibrils, but increased the granular and amorphous aggregates [28]. It is known that deposits of amyloid fibrils interact with metals, such as Cu^2+^ and Fe^3+^, and generate free radicals in the Fenton reaction, yielding H_2_O_2_. Externally applied antioxidant substances can help the body to fight free radicals. Sometimes, antioxidants can become pro-oxidant under certain conditions, such as in the presence of transition metals, thereby generating H_2_O_2_, as is the case with vitC at higher concentrations.

For this in vitro study, we followed amyloid fibril formation by human stefin B to study the effect of polyphenols on amyloid fibril formation (curcumin, quercetin, and resveratrol—as before [27]) in combination with vitamin C. A synergistic effect was observed for some of the polyphenols, which warrants further clinical and dietary studies.

## 2. Materials and Methods

### 2.1. Materials

2,2,2-Trifluorethanol (TFE) was purchased from Fluka, Thioflavin T (ThT) was obtained from Aldrich, and bis(sulfosuccinimidyl)suberate (BS3) was provided by ThermoFisher Scientific. Antioxidants were from Sigma-Aldrich (St. Louis, MO, USA). Other chemicals were from Sigma (St. Louis, MO, USA), Carlo Erba (Val de Reuil, France), Serva (Heidelberg, Germany), or Merck (Darmstadt, Germany).

### 2.2. Protein Expression and Purification

Human stefin B (stB wt) was isolated in recombinant form with Cys3 replaced with Ser [29]. DNA constructs were transformed into the BL21(DE3)pLysS strain of *E. coli*. Expression was induced with IPTG (final concentration 1 mM). Three hours after induction, cells were separated from the medium and lysed. Cell lysates were additionally purified by adding 4% polyethylenimine (PEI) and repetitively centrifuged. The stB wt was isolated from purified cell lysate by affinity chromatography on carboxymethylated (CM) papain Sepharose. Nonspecifically bound material was eluted with 0.01 M Tris-HCl containing 0.5 M NaCl at pH 8.0 stB wt was eluted with 0.02 M TEA buffer at pH 10.5. Additional purification was obtained by SEC on Sephacryl S-200 (Amersham Pharmacia Biotech, Buckinghamshire, UK) equilibrated with 0.01 M phosphate buffer, containing 0.12 M NaCl at pH 6.1. Purity was checked by SDS PAGE electrophoresis.

### 2.3. ThT Fluorescence

ThT dye fluorescence was measured using a Perkin-Elmer model LS 50 B luminescence spectrometer. Excitation was at 440 nm, and spectra were recorded from 455 nm to 600 nm. ThT dye was dissolved in phosphate buffer (25 mM, 0.1 M NaCl at pH 7.5) at 15 μM (A_416_ = 0.66). Fibrils were grown under mild conditions at pH ~5 (0.015 M acetate buffer, 0.15 M NaCl at pH 4.8) at room temperature; protein concentration was 34 μM. In order to accelerate fibril formation, fibrillation mixtures contained 10% *v*/*v* TFE. Then, 50 μL of the protein solution in which fibrils were growing was added to 570 μL of the ThT buffer just before the measurement. A fresh ThT probe was prepared daily. ThT fluorescence was measured using a 0.5 cm cuvette at 25 °C. Excitation and emission slits were set at 5 nm and 7 nm, respectively. Data were collected every 0.5 nm. Each measurement was performed at least twice in duplicates, and the mean value was presented. Kinetic data analysis was performed by sigmoidal curve fitting [27].

### 2.4. Transmission Electron Microscopy (TEM)

First, 15 μL of 34 μM protein solution was applied on a Formvar and carbon-coated grid. After 3 min, the sample was soaked away and stained with 1% (*w*/*v*) uranyl acetate. Samples were observed with a Philips CM 100 (FEI, Amsterdam, The Netherlands) transmission electron microscope operating at 80 kV. Images were recorded by Bioscan CCD or ORIUS SC 200 camera (Gatan Inc., Washington, DC, USA), using Digital Micrograph software (Gatan Inc., Washington, DC, USA). Two parallel grids were prepared for each sample, at least 10 grid squares were inspected thoroughly, and many micrographs were taken of each grid.

## 3. Results

### 3.1. Kinetics of the Fibrillation by stB in Presence of Curcumin and Vitamin C and Morphologic Characterization of the Fibrils at the Plateau Phase

In order to observe the eventual synergistic effects of polyphenolic antioxidants curcumin (Cur) and vitamin C (vitC) on amyloid fibril formation by stB, we measured ThT fluorescence as a function of time at various Cur concentrations while keeping vitC concentration constant, 0.5 mM. At this concentration, vitC alone most potently inhibits the amyloid fibril formation of stB [27].

**Figure 1:**
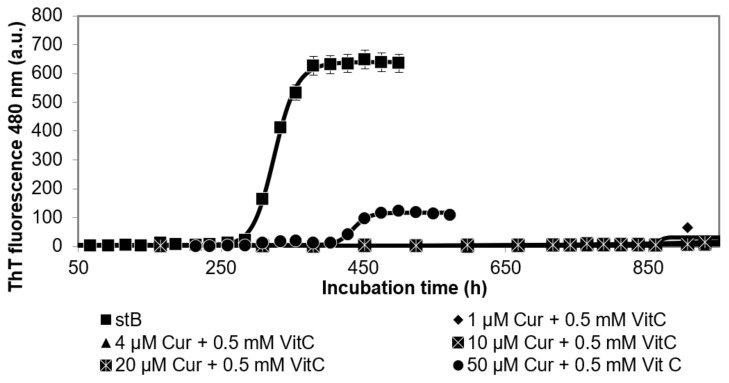
Time course of ThT fluorescence intensity following the amyloid fibril formation of stB in presence of curcumin (Cur). Different concentrations of Cur (as indicated) were added, while vitC concentration was kept constant at 0.5 mM.

Thus, the results of the kinetics measured by ThT fluorescence (Figure 1) indicated that fibrillation was absent at 1 µM Cur + 0.5 mM VitC, 4 µM Cur + 0.5 mM VitC, 10 µM Cur + 0.5 mM VitC, and 20 µM Cur + 0.5 mM VitC. Only at 50 µM Cur + 0.5 mM VitC did the fibrillation reaction occur as judged by ThT fluorescence, but after a prolonged lag phase of 400 h, which was 140 h longer than that for stB alone. However, the final ThT fluorescence at the plateau phase remained low (Figure 1). This prolongation of the lag phase was significantly longer than the increase of 100 h when 50 µM Cur alone was present in the fibrillation mixture together with stB, as shown before [27].

**Figure 2:**
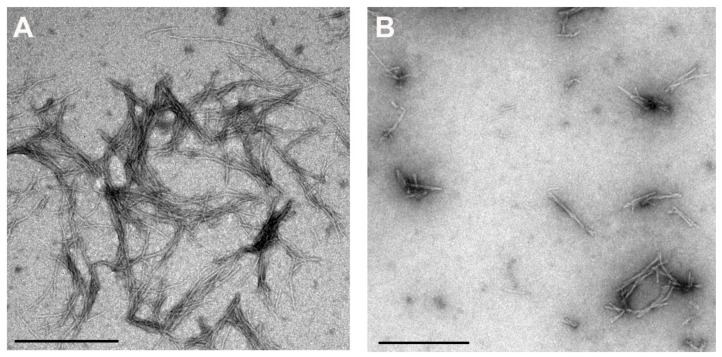
TEM images of stB fibrils at the plateau phase (**A**) (adapted from Ref. [27]) alone or (**B**) at 50 µM Cur + 0.5 mM vitC. Scale bars = 0.5 µm.

To observe the morphology of the fibrils produced by a combined effect of Cur and vitC, Transmission electron microscopy (TEM) was performed at 50 µM Cur + 0.5mM vitC (Figure 2), where amyloid fibrils are indicated. At all other concentrations of Cur + 0.5 mM vitC, fibril growth was completely inhibited. TEM results (Figure 2) show that there were indeed fewer amyloid fibrils, and they were of different morphology in comparison to stB alone; they seemed shorter and thicker.

### 3.2. Kinetics of the Fibrillation Reaction by stB in Presence of Resveratrol and Vitamin C and Morphology of the Fibrils at the Plateau Phase

**Figure 3:**
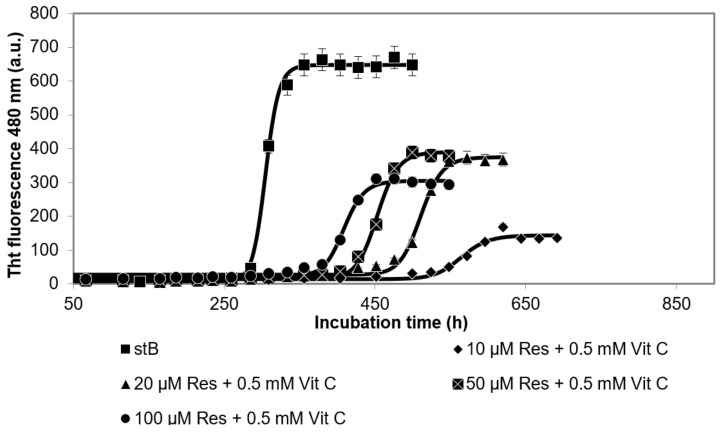
Time course of ThT fluorescence intensity following amyloid fibril formation of stB in the presence of resveratrol (Res). Different concentrations of Res (as indicated) were added, while vitC concentration was kept constant at 0.5 mM.

**Figure 4:**
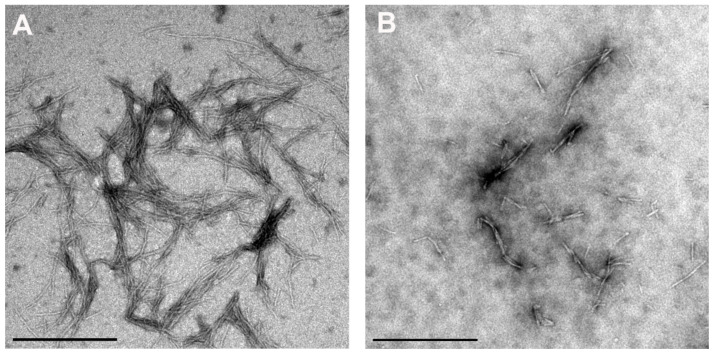
TEM images of stB fibrils at the plateau phase (**A**) alone or (**B**) at 100 µM Res + 0.5 mM vitC. Scale bars = 0.5 µm.

Results of the kinetics of the fibrillation reaction of stB measured by ThT fluorescence (Figure 3) showed that, at all concentrations of Res at a constant 0.5 mM vitC (10 µM Res + 0.5 mM vitC, 20 µM Res + 0.5 mM vitC, 50 µM Res + 0.5 mM vitC, and 100 µM Res + 0.5 mM vitC), the lag phase was prolonged in comparison to the lag phase of stB in the absence of antioxidants. Moreover, the intensity of ThT fluorescence in the lag phase was significantly decreased. Kinetics did not differ at 10 µM Res + 0.5 mM vitC in comparison to stB alone; neither the lag phase nor the intensity was changed at this concentration of Res. At 20 µM Res + 0.5 mM vitC, the lag phase was prolonged for 280 h; at 50 µM + 0.5 mM vitC, it was prolonged for 148 h; at 100 µM Res + 0.5 mM vitC, it was prolonged for 122 h. On the other hand, ThT fluorescence was significantly decreased in all three cases. TEM results confirmed stB fibrils at the plateau phase in the presence of 100 µM Res + 0.5 mM vitC (Figure 4).

### 3.3. Kinetics of the Fibrillation by stB in Presence of Quercetin and Vitamin C and Morphologic Characterization of the Fibrils at the Plateau

**Figure 5:**
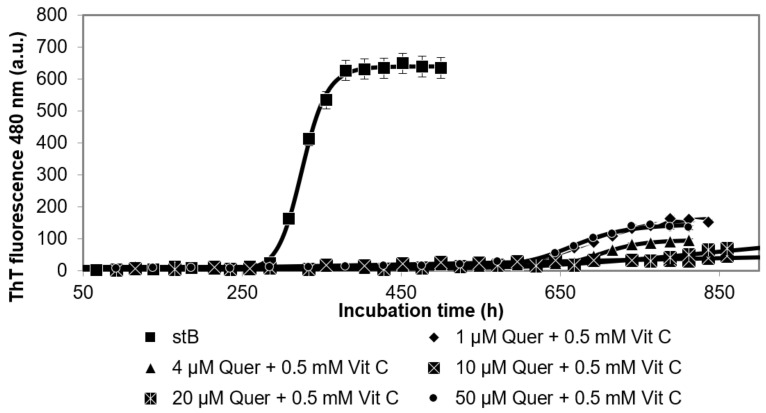
Time course of ThT fluorescence intensity following the amyloid fibril formation of stB in the presence of quercetin (Quer). Different concentrations of Quer (as indicated) were added, while vitC concentration was kept constant at 0.5 mM.

The time course, i.e., the kinetics, of fibrillation by stB as measured by ThT fluorescence (Figure 5) showed that, at all concentrations of Quer and additional 0.5 mM vitC (1 µM Quer + 0.5 mM vitC, 4 µM Quer + 0.5 mM vitC, 10 µM Quer + 0.5 mM vitC, 20 µM Quer + 0.5 mM vitC, and 50 µM Quer + 0.5 mM vitC), the lag phase was prolonged for about 450 h in comparison to stB without antioxidants. Furthermore, the intensity of ThT fluorescence at the plateau phase was drastically lowered. This effect was much stronger than the effect of 0.5 mM vitC alone.

**Figure 6:**
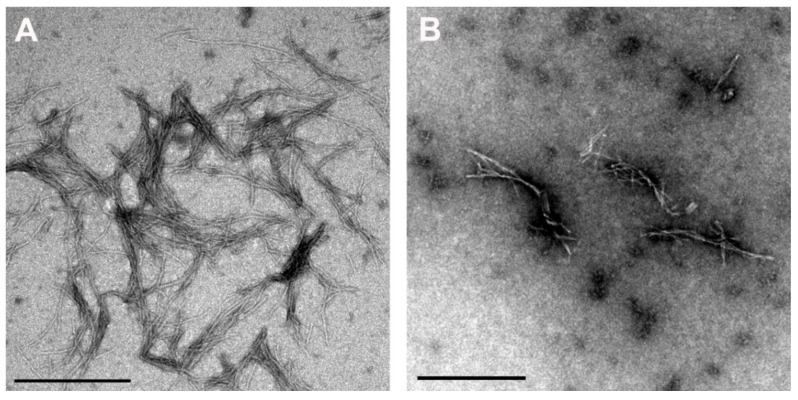
TEM images of stB fibrils at the plateau phase (**A**) alone or (**B**) at 4 µM Quer + 0.5 mM vitC. Scale bars = 0.5 µm.

TEM results confirmed the above observations, whereby the fibrils in the presence of 4 µM Quer and 0.5 mM vitC (Figure 6) were thicker and shorter, and their amount was less.

## 4. Discussion and Conclusions

The aim of this in vitro study was to evaluate the combined effect of different polyphenolic compounds and vitC on amyloid fibril formation by human stefin B (stB). We intended to test the hypothesis that polyphenols and vitC act synergistically. For that goal, we investigated the effects of different concentrations of resveratrol, quercetin, and curcumin while keeping vitC constant at 0.5 mM. This concentration of vitC was chosen as that which had the biggest inhibitory action on amyloid fibril formation by stB. Indeed, the results of ThT fluorescence against time confirmed some synergistic effects, but this did not hold for all polyphenolic compounds equally. The strongest effect was observed in the case of Cur (1 µM, 4 µM, 10 µM, and 20 µM) plus 0.5 mM vitC. The combined effect of curcumin and vitC was greater than either curcumin or vitamin C alone (as previously shown by Hasanbasic et al. [27]), which confirms synergy (Figure 1). The only exception was represented by 50 µM Cur with 0.5 mM vitC, where the combined inhibitory effect was between that of curcumin and vitC alone.

It is possible that the highest Cur concentration may have decreased the effect of vitC. TEM analysis (Figure 2) showed that the morphology of the fibrils (shorter and thicker) was changed at 50 µM Cur and persisted in the presence of 5 mM vitC (Figure 2). Both Cur and vitC inhibit the phase of nucleation, whereas they most likely bind to different binding sites of the β-pleated sheet of stB amyloid fibrils, which results in a synergistic effect.

In contrast to the combined effect of different concentrations of Cur and 0.5 mM vitC, different concentrations of Res (10 µM, 20 µM, 50 µM, and 100 µM) and 0.5 mM vitC did not show synergy. The time course of the fibrillation reaction by stB in the presence of the lowest 10 µM Res and 0.5 mM vitC was analogous to the kinetics of stB at 0.5 mM vitC alone, in terms of the length of the lag phase and intensity of ThT fluorescence [27]. Likely, even at the lowest Res concentration, vitC can still exhibit complete inhibitory action. At higher concentrations of Res, however, the lag phase was decreased (Figure 3). ThT fluorescence results were in accordance with TEM analysis, which showed that the morphology of amyloid fibrils at 100 µM Res and 100 µM Res + 0.5 mM vitC (Figure 4) was similar. In both cases, the fibrils were shorter and thicker than those formed by stB alone. This indicates that both antioxidants may bind to the same site on the β-pleated sheet of amyloid, with a distinction that vitC influences the phase of nucleation, while Res does not; instead, it influences the morphology of the mature fibrils. This is in accordance with other research, which showed that Res influences the amyloid fibril formation of Aβ peptide in such a way that it changes its morphology and renders previously toxic fibrils nontoxic [30,31].

Of interest, Quer and vitC also demonstrated a strong synergistic effect on the inhibition of amyloid fibril formation by stB. As previously shown by Hasanbasic et al. [27], Quer alone does not influence much the lag phase; instead, it decreases the final ThT fluorescence, whereas TEM data showed the accumulation of amorphous aggregates and fibrils of changed morphology, which are shorter and thicker [27].

Various concentrations of Quer (1 µM, 4 µM, 10 µM, 20 µM, and 50 µM) plus 0.5 mM vitC, by ThT fluorescence (Figure 5), showed stronger inhibition by prolongation of the lag phase than Quer or vitC alone. Intensity of ThT fluorescence decreased with Quer concentration (4 µM Quer, 10 µM Quer, and 20 µM Quer + 0.5 mM vitC) and was lower than 0.5 mM vitC alone. In accordance, TEM results of the sample 1 µM Quer + 0.5 mM vitC (Figure 6) confirmed fewer fibrils, which were shorter and thicker. There were no amorphous aggregates, as previously observed in presence of 1 µM Quer and 0.2 mM vitC alone. Thus, both ThT fluorescence and TEM confirmed a synergistic effect of Quer and vitC, which must, therefore, bind to different sites on the β-pleated sheet of stB amyloid fibrils.

Below, we explain why we use human stefin B as a good model protein to study transformation into amyloid, even though there have been no reports that this protein may be involved in a clear amyloid disease? Indeed, under normal physiological conditions, human stefin B, as a housekeeping gene, acts as an intracellular cathepsin inhibitor. It is localized in the cytosol, as well as in the nucleus [32]. It was found to be multimeric in cells [33,34], which suggests it may have alternative functions than cysteine protease inhibition. Its oligomers were shown to interact with cytoskeletal proteins [33] and to bind Aβ [35], suggesting a chaperone-like function [36,37]. An Italian group demonstrated that stefin B (cystatin B) is important in the physiology of the synapse [38] and is essential for proliferation and interneuron migration [39]. When cystatin B gene is mutated, it causes a rare progressive myoclonus epilepsy—EPM1 [40,41], spread in the Baltics and some parts of the Mediterranean. Most mutations are dodecamer repeats [42] in the gene’s promotor region; however, quite a number of missense mutants [43] prone to aggregate have also been reported [44]. EPM1 is a progressive myoclonus epilepsy with some features of neurodegeneration, which may be due to protein aggregation or insufficient clearance of the aggregates [45,46].

In contrast, human cystatin C is clearly involved in amyloid pathology. Its mutation L68Q causes a severe amyloid disease, hereditary cystatin C amyloid angiopathy (HCCAA). HCCAA is a rare autosomal dominant genetic disease observed in a small part of the population of Iceland. The amyloid deposits of the L68Q mutated CysC, which accumulate in the brain arteries, weaken arterial walls, which leads to repeated brain hemorrhages (mini-strokes) and may result in dementia and paralysis [47]. Cystatin C also binds Aβ [48,49,50] and was found to be neuroprotective, loaded in extracellular vesicles [51]. Importantly, cystatins are constituents of amyloid plaques [19].

Nonetheless, using stB as an amyloid-forming protein is reasonable and easier than cystatin C, which possesses two disulfide bonds and needs high T to undergo transition. Implications of our study in vitro for EPM1 disease are also obvious. A diet rich in polyphenols, such as curcumin and quercetin, together with modest vitamin C supplementation, could be put in place, and one would expect at least a mild positive outcome (at least, in some cases, depending on the phenotype).

## Data Availability

The data presented in this study are available in article.
