# Correction: Jahić Mujkić et al. Synergy of the Inhibitory Action of Polyphenols Plus Vitamin C on Amyloid Fibril Formation: Case Study of Human Stefin B. *Antioxidants* 2021, *10*, 1471

**DOI:** 10.3390/antiox13010056

**Published:** 2023-12-28

**Authors:** Alma Jahić Mujkić, Magda Tušek Žnidarič, Selma Berbić, Eva Žerovnik

**Affiliations:** 1 Department of Biochemistry and Molecular and Structural Biology, Jožef Stefan Institute, Jamova 39, 1000 Ljubljana, Slovenia; 2 Department of Biochemistry, Faculty of Pharmacy, University of Tuzla, Univerzitetska 1, 75000 Tuzla, Bosnia and Herzegovina; 3 Department of Biotechnology and Systems Biology, National Institute of Biology, Večna pot 111, 1000 Ljubljana, Slovenia; magda.tusek.znidaric@nib.si; 4 Jožef Stefan International Postgraduate School, Jamova 39, 1000 Ljubljana, Slovenia

In the original publication [1], there was a mistake in the legend for Figure 2. The image (A) is the same as that published before (Figure 8b) in [27], as the conditions for the control did not differ. The correct legend appears below. The authors state that the scientific conclusions are unaffected. This correction was approved by the Academic Editor. The original publication has also been updated.

**Figure 2:** TEM images of stB fibrils at the plateau phase (**A**) (adapted from Ref. [27]) alone or (**B**) at 50 μM Cur + 0.5 mM vitC. Scale bars = 0.5 μm.
